# Supplementary material for: The genome sequence of Hypericum androsaemum L., 1753 (Malpighiales: Hypericaceae)
Source: Wellcome Open Res. 2026 Jun 30;11:404. [Version 1] doi: 10.12688/wellcomeopenres.26904.1 (PMC13560572; doi:10.12688/wellcomeopenres.26904.1)
Supplement: Peer review history

# Reviewer report: Reviewer response for version 1

**DOI:** 10.21956/wellcomeopenres.29623.r160011

**Published:** 2026-08-19

**Authors:** Bimal K Chetri

**Affiliations:** 1 Department of Natural Sciences, Royal University of Bhutan Sherubtse College (Ringgold ID: 93080), Kanglung, Trashigang, Bhutan

The manuscript reports a flow-cytometry estimate of ~480 Mb (0.49 pg), a GenomeScope2 estimate of 402.58 Mb, and a final assembly size of 384.54 Mb. While such differences are not unusual, a brief comment explaining the discrepancy between the flow cytometry estimate and the assembled genome size would provide useful context for readers and aid interpretation of assembly completeness.

Are sufficient details of methods and materials provided to allow replication by others?

Yes

Is the rationale for creating the dataset(s) clearly described?

Yes

Are the datasets clearly presented in a useable and accessible format?

Yes

Are the protocols appropriate and is the work technically sound?

Yes

Reviewer Expertise:

Organellar genome study and plant flow cytometry

I confirm that I have read this submission and believe that I have an appropriate level of expertise to confirm that it is of an acceptable scientific standard.

# Reviewer report: Reviewer response for version 1

**DOI:** 10.21956/wellcomeopenres.29623.r161342

**Published:** 2026-07-31

**Authors:** Nils Stein

**Affiliations:** 1 Leibniz Institute of Plant Genetics and Crop Plant Research (IPK), Seeland, Germany

The authors report on a chromosome scale genome assembly of *Hypericum androsaemum, *endemic to the Britain and Ireland, as part of the DToL project, generating data by standardized protocols of the DToL project and following established data standards for DToL project assemblies. Based on the genome stats and the size estimates the results look as if the assembly represents a haploid assembly of a highly homozygous genome. Chromosome scale scaffold number coincides with the n=20 for the haploid set of chromosomes.

 The content of this data publication is straightforward. The quality of the HiC interaction plot is low and is difficult to really assess. It would be recommended if WOR would provide a webapplication for inspecting HiC interactionplots for all their published genomes.

Are sufficient details of methods and materials provided to allow replication by others?

Yes

Is the rationale for creating the dataset(s) clearly described?

Yes

Are the datasets clearly presented in a useable and accessible format?

Yes

Are the protocols appropriate and is the work technically sound?

Yes

Reviewer Expertise:

Plant genetics and genomics

I confirm that I have read this submission and believe that I have an appropriate level of expertise to confirm that it is of an acceptable scientific standard.

# Reviewer report: Reviewer response for version 1

**DOI:** 10.21956/wellcomeopenres.29623.r161345

**Published:** 2026-07-31

**Authors:** Himanshu Saini

**Affiliations:** 1 Adama Science and Technology University, Adama, Ethiopia

This manuscript presents a high-quality chromosome-scale genome assembly of *Hypericum androsaemum* (Tutsan), generated as part of the Darwin Tree of Life (DToL) project.

 The assembly comprises a total genome size of **384.54 Mb**, with **99.96%** of the sequence assigned to **20 chromosomal pseudomolecules**, together with assembled mitochondrial and plastid genomes. The study provides an important genomic resource for the Hypericaceae family, supporting future research in plant evolution, comparative genomics, conservation biology, and medicinal plant genetics.

 The manuscript is technically well prepared and follows the standard format of DToL genome notes. The assembly statistics indicate excellent sequencing and scaffolding quality, and the work makes a valuable contribution by providing the first or among the first chromosome-level reference genomes for this species.

 However, as a genome announcement rather than a comprehensive biological study, the manuscript contains limited biological interpretation. While this is acceptable within the journal's scope, several sections could be strengthened to improve the scientific value and long-term usefulness of the resource.

Are sufficient details of methods and materials provided to allow replication by others?

No

Is the rationale for creating the dataset(s) clearly described?

Yes

Are the datasets clearly presented in a useable and accessible format?

Yes

Are the protocols appropriate and is the work technically sound?

Yes

Reviewer Expertise:

Molecular Plant Breeding

I confirm that I have read this submission and believe that I have an appropriate level of expertise to confirm that it is of an acceptable scientific standard, however I have significant reservations, as outlined above.

# Reviewer report: Reviewer response for version 1

**DOI:** 10.21956/wellcomeopenres.29623.r161346

**Published:** 2026-07-21

**Authors:** Ruslan Kalendar

**Affiliations:** 1 University of Helsinki, Helsinki, Finland

This Data Note reports a chromosome-level reference genome assembly for *Hypericum androsaemum* (tutsan), generated within the Darwin Tree of Life (DToL) Project from a single specimen (ddHypAndr1) collected in Helston, Cornwall, UK. The assembly follows the well-established DToL pipeline: PacBio HiFi long reads assembled with Hifiasm, Hi-C scaffolding with YaHS, organelle assembly with OATK, decontamination with ASCC/BlobToolKit, and manual curation. The primary assembly spanned 384.54 Mb, with 99.96% of the sequence assigned to 20 chromosomal pseudomolecules (scaffold N50 19.81 Mb; contig N50 2.9 Mb; consensus QV 64.1; k-mer completeness 99.05%). A 165 kb plastid genome and four mitochondrial sequences have also been reported. All raw reads and haplotype assemblies were deposited in INSDC/ENA under BioProject PRJEB73427, and the software versions were fully tabulated.

 Overall, this is a well-executed, clearly written genome note describing a high-quality, openly available assembly that meets the Earth BioGenome Project (EBP) reference-quality contiguity and consensus-accuracy targets (6).C.Q64 against the recommended value of 6.C.Q40). This study provides a useful addition to the growing set of chromosome-scale plant reference genomes and the genomics of Malpighiales/Hypericaceae. The methods are thorough and reproducible, and the data availability practice is exemplary. I recommend indexing after the authors address a small number of points, the most important of which concerns the interpretation of the assembly in light of the documented ploidy history of the species.

**1. **The most substantive gap is the absence of any discussion on the chromosome number and ploidy background of *H. androsaemum*. This species has long been reported as 2n = 40, double the 2n = 20 of taxa in related sections, and has been interpreted either as an allopolyploid (amphidiploid) or as an autotetraploid that has undergone diploidization, with inheritance data showing diploid-like segregation (Robson & Adams, 1968; Olsen *et al.*, 2006; Trueblood *et al.*, 2010). This context is directly relevant to the three results in this study.

- The recovery of exactly 20 pseudomolecules was consistent with n = 20 (2n = 40). Stating the expected chromosome number explicitly and confirming that the assembly recovers the expected karyotype would strengthen the note and reassure the reader that no chromosomes are missing or fused.
- The BUSCO profile showed an elevated duplication rate (D = 14.5%) and a correspondingly reduced single-copy fraction (S = 80.5%). As shown in the authors’ Table 4, this row does **not** meet the stated BUSCO benchmark (S > 90%; D < 5%), even though every other EBP metric (contiguity, QV, k-mer completeness, and% assigned to chromosomes) is comfortably met. The polyploid ancestry of this species is a plausible biological explanation for the retention of duplicate copies. I would ask the authors to (i) acknowledge in the text that the BUSCO benchmark is not met and (ii) briefly discuss the most likely cause of retained homeologs from the polyploid history versus residual haplotypic duplication not resolved during curation (40 haplotypic duplications were removed).
- GenomeScope2 was run using a diploid model (p = 2, Figure 2). Given the diploidized inheritance, this is defensible, but the choice would benefit from a one-line justification in the context of the species' ploidy.

**2. **Three independent size estimates were obtained: flow cytometry (1C = 480 Mb), GenomeScope2 (402.58 Mb), and the assembled span (384.54 Mb). The assembly is therefore 95% of the k-mer-based estimate but only 80% of the flow cytometric estimate. A sentence on the likely nature of the unassembled fraction (e.g. collapsed or under-represented repetitive/heterochromatic sequence, rDNA arrays) would help readers judge completeness, particularly given the 32% repeat content estimated by GenomeScope.

**3. **The abstract and Genome sequence report describe "multiple mitochondrial genomes (4)" of 138.63, 75.57, 98.15, and 103.38 kb. Because plant mitochondrial genomes are frequently multipartite, it would be clearer to state whether these four sequences represent a single multipartite mitochondrial genome (multiple molecules/subgenomic circles of one mitogenome) or distinct/alternative assembled contigs. As written, "four mitochondrial genomes" can be interpreted as four separate organellar genomes, which is presumably not the intended meaning.

**4. **OATK used the *Salix dunnii* mitogenome (NC_058734.1) as a reference genome. *Salix* is a fellow member of Malpighiales but is phylogenetically distant from Hypericaceae; a brief note that this was the closest suitable high-quality reference available would be helpful.

**5.** *H. androsaemum* is often described as semi-evergreen rather than fully deciduous species. The authors may wish to check the wording in the Background against their preferred flora.

**6. **A few minor points that do not affect the science: the second author’s affiliation contains a duplicated "England, England"; the collection longitude for Helston should appear as a negative (west) value in the Methods to match Table 1; and the estimated coverage ("approximately 41 coverage") should read "41×".

Are sufficient details of methods and materials provided to allow replication by others?

Yes

Is the rationale for creating the dataset(s) clearly described?

Yes

Are the datasets clearly presented in a useable and accessible format?

Yes

Are the protocols appropriate and is the work technically sound?

Yes

Reviewer Expertise:

bioinformatics genome comparison

I confirm that I have read this submission and believe that I have an appropriate level of expertise to confirm that it is of an acceptable scientific standard, however I have significant reservations, as outlined above.

# Reviewer report: Reviewer response for version 1

**DOI:** 10.21956/wellcomeopenres.29623.r160012

**Published:** 2026-07-21

**Authors:** Peng-Fei Ma

**Affiliations:** 1 Chinese Academy of Sciences, Kunming, China

The genome of tutsan ( *Hypericum androsaemum*) was sequenced and assembled in this study.

 Full details were given for the genome sequencing, assembly, and evaluation. And it is a high-quality genome assembly.

 However, the information of basic chromosome numbers of this species was not introduced and how the authors know to assemble the genome into 20 chromosomal pseudomolecules?

Are sufficient details of methods and materials provided to allow replication by others?

Yes

Is the rationale for creating the dataset(s) clearly described?

Yes

Are the datasets clearly presented in a useable and accessible format?

Yes

Are the protocols appropriate and is the work technically sound?

Yes

Reviewer Expertise:

Plant genomics and evolution

I confirm that I have read this submission and believe that I have an appropriate level of expertise to confirm that it is of an acceptable scientific standard.
